# Probe Request Based Device Identification Attack and Defense

**DOI:** 10.3390/s20164620

**Published:** 2020-08-17

**Authors:** Xiaolin Gu, Wenjia Wu, Xiaodan Gu, Zhen Ling, Ming Yang, Aibo Song

**Affiliations:** 1 School of Cyber Science and Engineering, Southeast University, Nanjing 211189, China; xiaolin_gu@seu.edu.cn; 2 School of Computer Science and Engineering, Southeast University, Nanjing 211189, China; xdgu@seu.edu.cn (X.G.); zhenling@seu.edu.cn (Z.L.); yangming2002@seu.edu.cn (M.Y.); absong@seu.edu.cn (A.S.)

**Keywords:** device identification, 802.11ac network, probe request, deep learning, stream cipher

## Abstract

Wi-Fi network has an open nature so that it needs to face greater security risks compared to wired network. The MAC address represents the unique identifier of the device, and is easily obtained by an attacker. Therefore MAC address randomization is proposed to protect the privacy of devices in a Wi-Fi network. However, implicit identifiers are used by attackers to identify user’s device, which can cause the leakage of user’s privacy. We propose device identification based on 802.11ac probe request frames. Here, a detailed analysis on the effectiveness of 802.11ac fields is given and a novel device identification method based on deep learning whose average f1-score exceeds 99% is presented. With a purpose of preventing attackers from obtaining relevant information by the device identification method above, we design a novel defense mechanism based on stream cipher. In that case, the original content of probe request frame is hidden by encrypting probe request frames and construction of probe request is reserved to avoid the finding of attackers. This defense mechanism can effectively reduce the performance of the proposed device identification method whose average f1-score is below 30%. In general, our research on attack and defense mechanism can preserve device privacy better.

## 1. Introduction

Internet of Thing (IoT) technology develops rapidly in recent years. It is reported that the global IoT device market is expected to reach $5.1 billion by 2025 [1]. It is well known that the Wi-Fi device is a major part of IoT devices. As a result, more and more researchers begin to pay attention to Wi-Fi security issues with the popularity of Wi-Fi devices. There is a high possibility for emitted radio frequency signals and transmitted frames to be eavedropped by malicious attackers due to the open nature of Wi-Fi. Physical signals and Media Access Control (MAC) frames can be used to identify a device, and the attacker can further track the device trace and analyze device’s vulnerabilities with CVE database [2], which can exploit user’s sensitive information. More specifically, distinguishing features which are strongly related to the hardware such as Central Processing Unit (CPU), network interface card, microphone, or the software such as devices’ driver, and website browser can be extracted and they can be used to conduct device identification.

Nowadays, the mainstream solutions to Wi-Fi device identification are classified into two categories: physical layer identification method and MAC frame identification method. As for physical layer identification method [3,4], it normally analyzes the Radio Frequency (RF) signal emitted from Wi-Fi network card and extracts physical differences such as imperfect oscillator and imbalance I/Q, which act as device physical features. The physical features are continuous variable so that they usually have large information entropy which avoid collision among different device fingerprints. However, this method usually depends on high-cost Software Defined Radio (SDR) equipment to collect the RF signals. Compared with the physical layer identification method, MAC frame device identification method [5,6,7,8,9,10] is relatively easy to implement because MAC frames in air can only be captured by a commerical network card which supports monitor mode. The probe request is often adopted in the MAC frame identification method. The reasons are as follows: firstly, the probe request is transmitted by the client device. Secondly, it is sent in clear text. The probe request contains a unique MAC address to identify the device. However a MAC address randomization will invalidate the MAC address of a transmitter [11]. Meanwhile, some fields like Service Set Identifier (SSID) list and vendor information can not be selected as features because they are possibly modified by users. Therefore, the existing MAC frame identification methods select other device-related features such as implicit identifiers from probe request to perform device identification. The combination of implicit identifiers in a probe request frame can be used as a device fingerprint to a certain extent. However, there are some disadvantages in the previous identification methods. In the first place, the most previous methods are only applicable to 802.11 b/g/n devices but not to 802.11 ac devices for Very High Through (VHT) information is not taken in consideration. In the second place, the previous methods based on the probe request are incapable of dealing with the random variations of values in frame body fields. On these grounds, we propose the 802.11ac device identification based on deep learning which can conduct the auto-feature selection.

Under the analysis of device identification technology, it has been found that MAC address randomization is not effective enough. Through the implicit identifiers in the probe request, attackers can still identify the device and steal users’ device privacy. At the same time, the usability of the probe request is required to be ensured because Access Point (AP) needs to know parameters of the terminal to ensure the normal operation of the 802.11 protocol. Therefore both the anonymity and usability of probe request need to be ensured. The regular privacy-preserving methods such as K-anonymization or differential privacy can not be applied in the probe request because it usually adds noises to conceal a single record, while our purpose is to obfuscate the training model. Thus, these two are completely different. In order to solve this kind of specific privacy threat, identifier-free approaches are proposed [12,13]. Identifier-free approaches encrypt all parts of a packet such as MAC header and payload. It can make plaintext and ciphertext different. However, these methods are accompanied with some disadvantages. First of all, identifier-free methods normally use public-key cryptography or block cipher in symmetric cryptography, whose computation cost is greater than that of stream cipher. Furthermore, the block cipher operates on a fixed length of bits and it possibly extends the least blocks with padding bits. It indicates that the encrypted MAC header is possibly longer than the original one, causing the encrypted MAC header to lose its original frame structure and increase the likelihood of probe request encryption being found by the attacker. As a result, the stream cipher in symmetric-key algorithm is chosen to obfuscate the value part of frame body in MAC header and remain the original frame structure to avoid the exposure of defense mechanism to attackers.

The major contributions of our work are summarized as follows:

- The general structure and fields of 802.11ac probe request are analyzed. An explanation of fields in frame body which are closely related to the device is given. These fields are proved to be used as a device fingerprint.
- A device identification method based on deep learning to select features automatically is studied and designed. It is aimed at overcoming the challenges brought by the random changes and fixed feature selection in 802.11ac probe request frame fields.
- An efficient mechanism against the attack on the basis of device identification is proposed. The stream cipher is used to hide the content of probe request for the purpose of protecting device privacy. In order to decrease the possibility that the attacker may find encrypted probe request frames, we only encrypt the value parts in MAC frame body to reserve the MAC header construction with stream cipher.
- We conduct experiments to evaluate our attack method and defense mechanism respectively. As for the attack method, the results show that average precision, recall and f1-score of our proposed device identification method exceed 99%. For the defense method, our proposed protection mechanism against the attack reduces the average precision, recall and f1-score of device identification to about 36%, 30% and 25% respectively.

The remainder of the paper is organized as follows. Section 2 reviews the relevant existing works. The device identification and its evaluation are presented in Section 3 and Section 4. Section 5 and Section 6 present the defense mechanism against the attack based on device identification and its evaluation. In Section 7, conclusions and discussions about future directions are presented.

## 2. Related Works

In this section, the related works about the device identification for attack and defense methods are presented.

### 2.1. Device Identification

There are a lot of research works about device identification in recent years. Physical features are used in device identification. Under the analysis of the deterministic errors in the calibration process, Zhang et al. [14] infer the per-device factory calibration data from the output of gyroscope, accelerometer and magnetometer. In addition to the motion sensors, the imperfection in embedded Acoustic Components can be used as the device fingerprints [15]. Tiny differences can be seen among magnetic signals radiated from different CPUs [16]. Li et al. [17] point out that the 3D printers can be identified by observing the differences of banding and attachment textures in different products due to the different manufacturing processes of the feeder, positioner and hot end. Hua et al. [4] extract carrier frequency offsets (CFOs) as the Wi-Fi card fingerprints from Channel State Information which refers to channel property of the current communication link. On the basis of Hua’s work, Liu et al. [3] take the I/Q imbalance into account and adopt the basic matrix operation to reduce the time overhead instead of the least square method. These methods based on the physical features usually require specialized equipment for data collection. Moreover, the physical features are specific and can only be applied to a few devices. Besides, other works focus on the features from network layer and web browser. Miettinen et al. [18] find that various IoT devices generate different network traffic sequences in the initial phases when they start to communicate with the cloud server. In the web browser fingerprinting, HTTP headers, JavaScript [19] and Cascade Style Sheets (CSS) [20] are proven to be effective in device identification. These features have more available dimensions, but they are easily to modified by the users or even attackers.

802.11 MAC frame is closely related to the underlying architecture of the device. In comparision with RF signals which are extracted from the professional and high-cost equipment (such as USRP), 802.11 MAC frame can be easily collected by commercial Wi-Fi cards in monitor mode. As long as vendors meet the requirement of the 802.11 standard, they can have their own implementations in the process of dealing packets. Franklin et al. [5] use the transmitting rate of the probe request frames as a differential feature. The large delays between cycles of probe request frames’ transmission can also form different clusters as device features [6]. Waltari et al. [7] analyze the probing behavior of mobile devices among multiple channels. Apart from the pattern of packet’s transmission, the devices can show diverse reactions upon the receipt of the carefully constructed packets whose fields are non-standard or unusual combinations [8]. In addition, values of the fields in MAC frame can be combined to form the fingerprints. Pang et al. [10] indicate that with regard to a device, its field combination is unlikely to change. The previous methods based on probing behavior is required to collect a large number of packets to calculate the average number of intervals and counts. Moreover, the normal state of the device is destroyed by the method based on the response of active probing. Above all, these methods based on 802.11 MAC fields are only associated with the device configuration, so they are relatively effective. However, most previous works based on MAC fields are unable to be directly applied to 802.11ac device identification because random changes can be seen in some fields in 802.11ac devices, and compared with 802.11 b/g/n, 802.11ac standard adds fields related to very high throughput characteristics. Robyns et al. [9] analyze the per-bit entropy of fields in probe requests of 802.11ac. They take the SSID list and vendor information as the candidate features. However, decided by the configuration of the upper layer, these features are unreliable. Therefore, we address the problems brought by random changes in the fields, and propose the 802.11ac device identification method based on deep learning in this paper.

### 2.2. Attack and Defense

Device identification can be used to track users or search vulnerabilities according to the device types in the CVE database in preparation for conducting the further attacks. The current defense methods against the attack of device identification in link layer are mainly divided into two categories which are pseudonym and identifier-free methods. The pseudonym method is used to obfuscate the MAC address which is an explicit identifier. Jiang et al. [21] use a well-known address to request the address pools to frequently change local MAC addresses. Fan et al. [22] take the reserving hash chain as a device identifier and determine an optimal number of hash operations which are aimed at conducting hash-chain re-synchronization in case of packet loss. The pseudonym method only removes the explicit identifier which leaks implicit information about 802.11 devices. Armknecht et al. [13] think RC5 is a perfect cipher and use origin or destination address as initial vectors to make ciphertext variable with RC5 every time. Greenstein et al. [12] design an 802.11-like protocol, which uses AES cipher to encrypt management and data frame to remove explicit and implicit identifiers. These identifier-free methods can erase all identifiers of the message, but also make the message lose its original structure in appearance. This will increase the possibility of the attacker to find the device defense strategy. In addition, the previous methods adopt block cipher or asymmetric encryption which have larger computation costs than that of the stream cipher. Therefore we propose a novel stream cipher-based defense mechanism against the attack which based on 802.11ac device identification.

## 3. Device Identification

Threat model of device identification is introduced in the first place. Then we analyze the 802.11ac protocol in detail. In the end we propose the device identification method based on deep learning.

### 3.1. Threat Model

**Figure 1:**
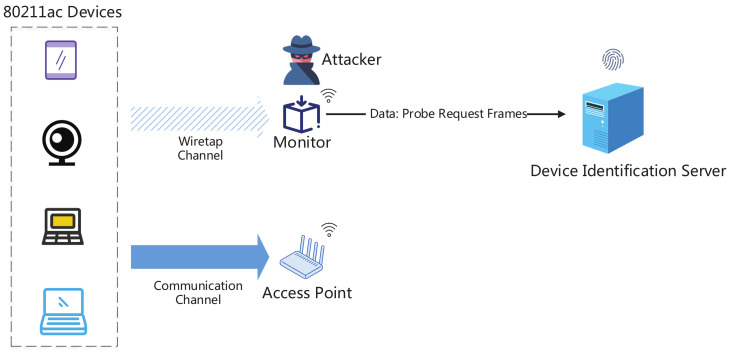
Threat model.

At the workplace, users usually own diverse mobile devices, which always send probe request to discover the existing nearly APs in an active manner. Any mobile devices including an attacker’s device can get the channel state information from the preamble and decode the entire probe request. In Figure 1, 802.11ac devices send probe request frames which include the device capabilities in wireless transmission to let AP know essential parameters of client devices. At the same time, a monitor is placed by an attacker in his interest region to capture the probe requests sent from 802.11ac devices in wiretap channel. Then the collected packets are transmitted to the remote server for device identification. For attackers, they can extensively collect probe requests sent by previously determined type of devices for model training and store the training model in the remote device identification server. After the type of the target device is identified, the attacker can find the vulnerability of the corresponding device by searching vulnerability databases like CVE [2], NVD [23] database in preparation of further invasion. The type of the targeted device is assumed to be in the device type list which have been collected in advance. Therefore, the Identification of unknown device types is out of scope.

### 3.2. 802.11ac Frame Analysis

We first analyze the basic structure of 802.11ac probe request and find some fields which have strong relationships with Wi-Fi capabilities.

**Figure 2:**
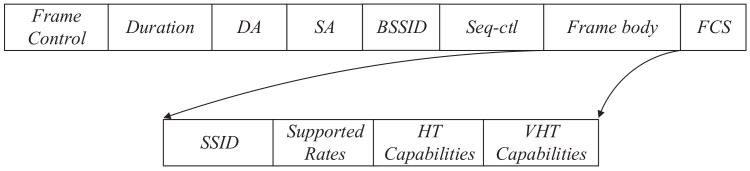
Basic structure of 802.11ac probe request frame.

Figure 2 presents a simplest frame structure of 802.11ac probe request which conforms to the 802.11ac standard [24]. It can be seen that only frame body varies with types of Wi-Fi devices which includes information elements and fixed fields, while the remaining part does not contain valid information about the device. Frame control field includes the frame type. And duration indicates how long the frame and its acknowledgment frame are going to occupy the channel. With regard to the probe request frame, the duration is always 0 because the AP does not reply to the station with ack frame [25]. The source address (SA) can be easily modified by open source network tools. The destination address (DA) is a fixed broadcast address ff:ff:ff:ff:ff:ff. As shown to all, the Seq-ctl represents the sequence number of a frame whose cycle is 4096. Thus, it increases as a probe request frame is sent. The frame check sequence (FCS) is responsible for checking the completeness of frame, which cannot be used as a feature.

On the basis of 802.11b/g/n standards, 802.11ac standard adds contents about VHT capabilities and the fingerprint’s capacity is expanded. The VHT capabilities field contains VHT capability info, VHT supported MCS and RX/TX MCS map, which shows the protocol operations supported by 802.11ac devices. It is found that most of the information is associated with physical capabilities of the Wi-Fi card chip. The physical capabilities are divided into 3 categories in Table 1, which are antenna, data rate and bandwidth.

Probe request of common commercial devices are collected and investigated in the market. For the VHT capabilities fields, they usually keep stable in the same device for a long time but display differences among some devices. Therefore, featured in stability and diversity, the VHT capabilities field can be used as a fingerprint. We carry out a simple experiment about device Redmi K20 pro from 14 October 2019 to 28 October 2019. Through the experiment, it is found that VHT capabilities info, VHT Support MCS and RX/TX MCS map are 0x3391f9fa, 0x030c and 0xfffa which keep stable. In addition, the probe request frame transmitted from usb Wi-Fi adapter Edimax ac-1200 has VHT capabilities info field whose value is 0x33c031a0. In comparision with the frames transmitted by two types of Wi-Fi chipset, it is concluded that the VHT capabilities field shows a different characteristic to some extent.

It seems that string perfect matching is a simple and effective method. However, some bits in the probe request have been found to change randomly among some devices. From the perspective of a malicious attacker, noises in the probe request frame hinder the performance of 802.11ac device identification. For example, the fields, beamformee STS capability and number of Sounding dimensions, are 3 bits and their maximum are 7. It is observed that the values in both fields change randomly within 4 in iphone X. In addition, the RX/TX MCS map varies from 0xfffa to 0xfffd randomly. It is supposed that some Wi-Fi chip manufacturers add the random noises into the probe request with the purpose of preventing the malicious attackers from exploring the users’ trace [26]. Next, an introduction on how to deal with the random changes of field values in Section 3.3 is provided. In addition, since many existing APs only support 802.11n protocol, most of the commercial network cards are required to be compatible with 802.11n. Therefore it makes probe request frame include some useful fields related to 802.11n standard protocol. And the extended capabilities are also affected by the OS and driver configurations in the probe request. All fields of 802.11n, 802.11ac and extended capabilities will be considered as an implicit fingerprint in order to gain a better performance in device identification.

### 3.3. Identification in Deep Learning

#### 3.3.1. Data Pre-Processing

A probe request frame normally includes the source MAC address, vendor specifics and SSID. Source MAC address and vendor specifics are explicit identifiers of wireless devices. Since SSID and the device are closely related, the SSID list in a probe request can also be regarded as a device fingerprint [27]. Since values of the three fields can be modified by users easily, these fields should be masked. According to 802.11ac standard, the source MAC address field whose length is 6 bytes, is placed in a fixed position. The vendor specifics and SSID belong to the information elements which consist of type id, length and variable-length value. Since the frame body also contains other items such as the HT/VHT capability information, every element needs to be parsed in accordance with the type-length-value structure. As Algorithm 1 shows, the corresponding bits of the source MAC address, vendor specifics and SSID field are located in a probe request frame, and their values are set to 0.

**Algorithm 1:** Probe request preprocessing

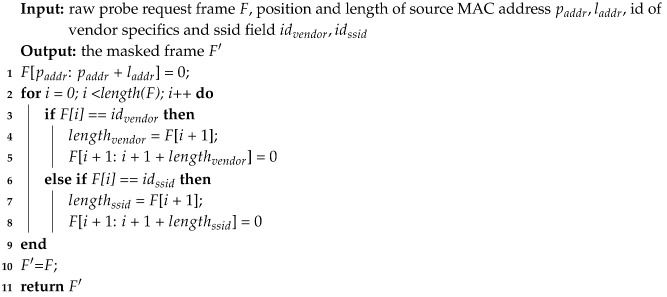



#### 3.3.2. The Construction of Neural Network

**Figure 3:**
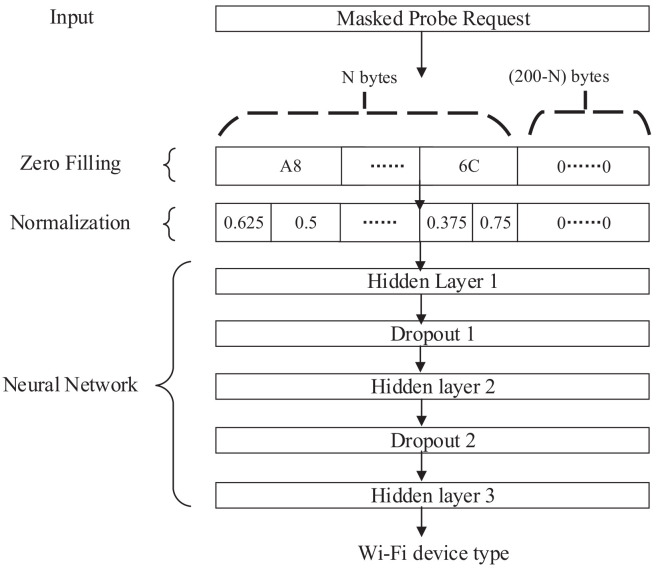
Construction of neural network.

The neural network is chosen as a classifier of device identification to extract features automatically due to the random changes in the probe request. A neural network has an advantage that features can be derived automatically from every bit in the probe request instead of manual feature selection. Compared with the picture and text data which have complicated structures, the probe request frame follows certain specifications of 802.11 protocol, so MLP (Multilayer Perception) is chosen as the deep learning method which has a relatively simple structure. As shown in the Figure 3, the size of the probe request is variable. And in actual measurements, its size is less than 200. The frame is padded with zero until its size reaches 200 bytes. Zero filling is used to enable the probe request to maintain the same length which meet the requirement of the MLP input format. By converting hexadecimal values in 4 bits and averaging them, we extract a 400-dimension vector and put it into the MLP to train a device classifier for device identification. In order to balance performance and computation costs, a light-weight neural network based on MLP is constructed. Under the guidance of the rules of thumb [28], the number of hidden layers is set to 3, and the number of neurons in each hidden layer is set to 128. Meanwhile, in order to prevent overfitting, the dropout is added between hidden layers and the dropout value to 0.3.

## 4. Evaluation of Device Identification

### 4.1. Experiment Setup

Experiments are conducted to evaluate the performance of our proposed device identification method. In order to evaluate the performance of 802.11ac device identification, a room with an area of 70 square meters located in the School of Computer Science and Engineering of Jiulonghu Campus of Southeast University is used.

- **802.11ac Devices**: 20 different types of devices are adopted as shown in Table 2. The uppercase letters O, D, C represent OS, driver and chipset types.
- **Monitor**: A Dell OptiPex 3600 mini workstation with usb wireless card Comfast CF-9391AC uses an open source tcpdump to capture 802.11ac probe request frames.
- **Device Identification Server**: A Dell desktop is adopted as a device identification server which preprocesses the packets, train device type neural network and identifies the corresponding type of a targeted device.

### 4.2. Performance Metrics

The standard classification metrics including precision, recall and f1-score are used as the performance metric of Wi-Fi device identification [16].

- **Precision**: For a certain device type, it represents the proportion of true positive samples among samples that are predicted to be positive.
- **Recall**: For a certain device type, it represents the proportion of true positive samples among all positive samples.
- **F1-score**: *2 × (precision × recall)/(precision + recall)*

### 4.3. Approach Based on Deep Learning

The performance of our proposed method based on deep learning is proposed. 4000 probe request frames sent by each one of 20 Wi-Fi devices are collected. All samples are split into training, validation and test sets according to the ratio of 6:2:2, which are used to train and evaluate the previous neural network model proposed in Section 3.3.2.

**Figure 4:**
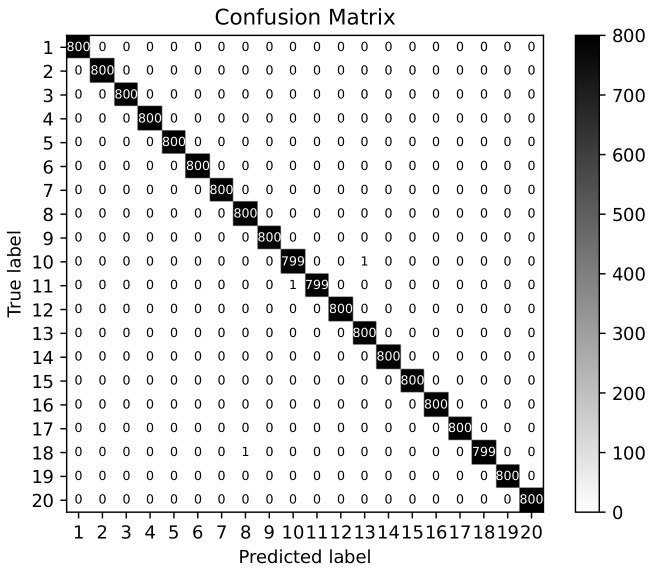
Device identification in matrix confusion.

Figure 4 shows that the identification precision of 17 devices reaches 100%. However, some samples of the remaining 3 devices are misclassified, as shown in Table 3. With the combination of the Table 2 and Table 3, it is found the misjudged devices have some certain similarities. From our point of view, the implicit identifiers in the probe request frames are relatively similar owning to the proximity of OS, driver or chipset. Therefore, fingerprint conflicts possibly occur because of random changes in field values. However, the average precision, recall and F1-score are still 99.98%, 99.98% and 99.98% shown in Table 4. FAR(False Acceptance Rate) is 0.001% and FRR (False Rejection Rate) is 0.02% which are also two metrics of the device identification [29]. It proves that our method is effective and robust in the case of random changes in field values.

### 4.4. Comparative Experiment

**Figure 5:**
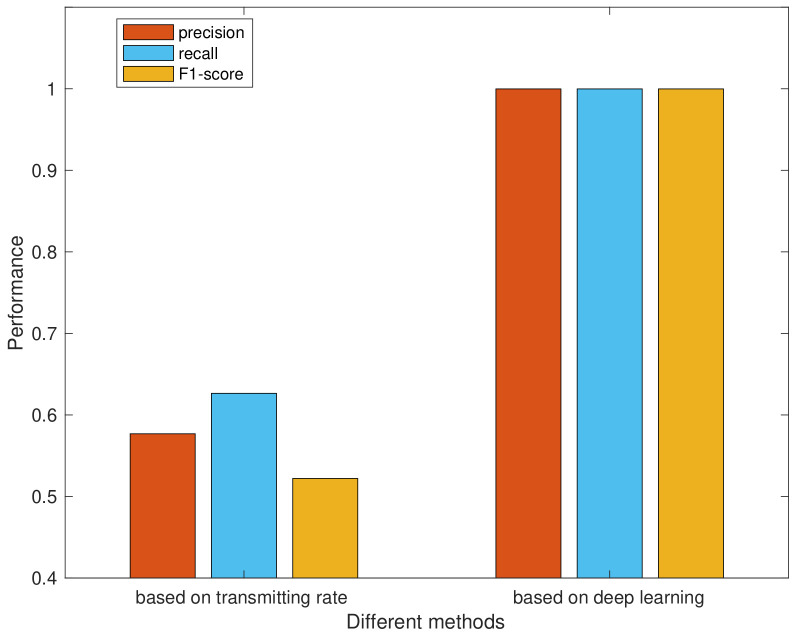
Performance of device identification methods.

Next, the performance of our proposed method is compared with the method based on transmitting rate. As Figure 5 shows, the average precision, recall and F1-score of method based on transmitting rate are only 57.69%, 62.65% and 52.21% respectively. It is worse than our proposed approach in the performance of device identification. According to our further studies, it is found that the rate of packet transmission is affected by the following factors in the experiment.

- **Packet Loss:** Some signals carrying messages can not be correctly demodulated by monitor sniffers because of the influence brought by random noise in the wireless channel. As a result, packet transmitting rate reflected in the monitoring point is lower than the actual packet transmitting rate of Wi-Fi devices.
- **Device Status:** The transmitting rate of packets is also affected by the current running status of Wi-Fi devices, such as antenna usage and bluetooth state [30].
- **Users’ operation habits:** On the basis of our findings, some mobile devices send probe request frames when users open the network selection interface of OS in practice. The Wi-Fi device also sends probe requests actively when the user chooses to connect to the AP. Undeniably, users have their own habits in using the Wi-Fi network, which will change the transmitting rate of original probe request.

In contrast, our method is based on the fields of the probe request frame, not easily affected the factors mentioned above. Thus, our method can achieve better performance than the method based on transmitting rate.

## 5. Defense Mechanism

In order to reduce risks caused by the attack based on 802.11ac device identification, our goal is to design an effective defense mechanism to preserve the probe request frames’ privacy while ensuring the usability of the probe request frames.

### 5.1. Security Requirements

Our basic idea is to obfuscate the implicit identifiers which is leaked during wireless communications and to make the real content received by AP. The security requirements which a defense mechanism should achieve are listed below:

- **Anonymity**: The proposed defense mechanism can conceal the implicit identifiers in the probe request frames for anonymity of the 802.11ac device type. 802.11ac device type can be identified with the above proposed identification method.
- **Structure Identifiability**: The proposed defense mechanism can ensure the identifiability of probe request frame structure. The structure identifiability indicates the structure of probe request frame is remained to avoid the awareness of adversaries.
- **Usability**: The proposed defense mechanism can ensure the availability of probe request frames, which is able to ensure the association and authentication operate normally between client and AP.
- **Unlinkability**: The proposed defense mechanism can make probe request frames unlinkable, which are sent out at different times. Although the linkability between probe request frames does not cause the privacy leakage of the 802.11ac device type itself, the adversary can track the targeted devices so as to choose a public region without security supervision. The targeted device is easily accessible so that the device type will be obtained offline.

### 5.2. Frame Encryption

**Figure 6:**
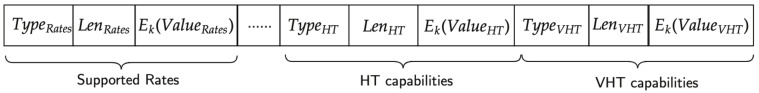
Structure of encrypted frame body.

According to Section 3.2, the implicit identifiers of probe request are in the frame body. As shown in Figure 6, the probe request is modified to meet security requirements within the defense mechanism: anonymity and structure identifiability. The fields in frame body are encapsulated in TLV(Type-Length-Value) format and the value part in fields is encrypted with the stream cipher. In short, the encryption algorithm is responsible for obfuscating the value part of fields in frame body asked by requirement of anonymity. Since the length of input and output are consistent in stream cipher, the encrypted value part still conforms to frame structure. As a result, the stream cipher can be applied into the value part in fields asked by requirement of structure identifiability. In this paper, chacha20 stream cipher [31], with secure and fast performance in the Chrome browser and Android is chosen. $E_{k}\left(value\right)$ represents the value is encrypted by a key *k* in chacha20 stream cipher. $D_{k}\left(value\right)$ represents the value which is decrypted by a key *k*. In addition, $E_{k}(probe$request) is an indication that value parts corresponding to the field types in the type list are encrypted in the probe request frame. The field types here are decided by AP because it has a strong sense of security in the area where it is located. Field types are contained in the beacon frame from AP.

### 5.3. Procedure of Defense Mechanism

The same key in one packet exchange for encryption and decryption needs to be generated in order to meet the system requirement of usability. Different keys are adopted to generate different ciphertexts in different rounds to ensure the frames’ unlinkability. Particularly, different keys also make the probe request difficult to be decrypted by attackers. Therefore diffle-hellman is leveraged to exchange a common key, which is used to act as random seed placed into a pseudo random number generator to generate a series of keys at the same time.

**Figure 7:**
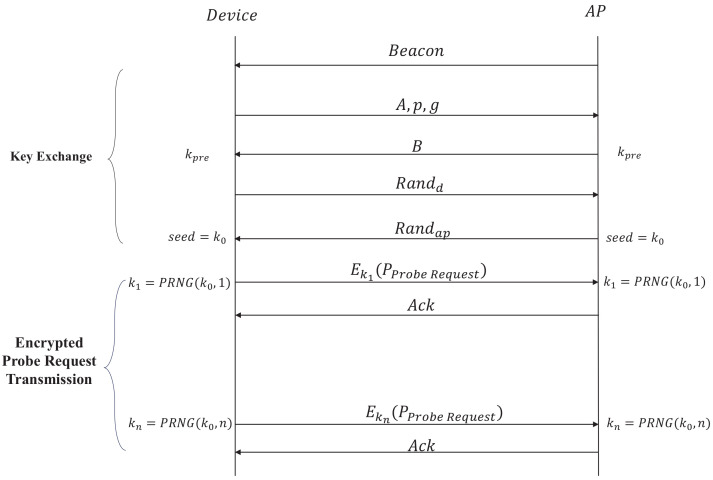
Frame exchange in defense mechanism.

As shown in Figure 7, the defense mechanism is divided into two phases: key exchange and encrypted probe request transmission. The common key is generated in the key exchange and encrypted probe request is sent in encrypted probe request transmission. The following part gives a brief introduction to the sending and receiving process of the client side and AP side.

- **Client Side**: When the client receives a beacon from AP which supports our proposed mechanism, it enters the key exchange phase and generates a secret integer *a*. Next, modulus *p* and base *g* are chosen to calculate public parameter *A*. As a matter of fact, the essential parameter *A*, *p* and *g* are sent to the AP for peer key negotiation. When the client received the public parameter *B* from AP, it can generate a key $k_{pre}$. In order to enhance the randomness of the key which acts as a seed in PRNG (Pseudorandom Number Generator), the key generation in TLS 1.2 protocol which exchanges individual random number is referred to. The client sends a random number ${Rand}_{d}$ and receives another number ${Rand}_{ap}$. Then the client uses $k_{pre}$, ${Rand}_{d}$, ${Rand}_{ap}$, client mac address ${MAC}_{d}$ and AP mac address ${MAC}_{ap}$ with PRF [32] applied in TLS to generate $k_{0}$ which has a good randomness. As for encrypted probe request transmission, $k_{0}$ is used as the seed of PRNG, so the client and AP can generate same random numbers synchronously. Besides, there are different random numbers in different rounds as keys of probe request encryption. This ensures that the frame body of the probe request can be decrypted correctly by AP, and the encrypted probe requests varies in different round of different frame exchanges. $PRNG(k_{0},n)$ represents that PRNG generates the *n*th key with seed $k_{0}$ and the round *n*. Here Mersenne Twister is used as PRNG which is widely used by many function libraries. Finally the client encrypts the probe request as described in Section 5.2. In addition, the sum of ${Rand}_{d}$ and ${Rand}_{ap}$ as the parameter nonce of chacha20 is assigned. The implementation detail is shown in Procedure 1.
- **AP Side**: The AP sends beacon frames to announce its existence constantly. When it receives essential parameters and random number from the client, the pair key $k_{0}$ is generated. Similar to the sending and receiving process of the client, the AP also uses the same PRNG to generate the key in order to decrypt the encrypted probe request. The implementation detail is shown in Procedure 2.

**Procedure 1:** Frame interaction proceduce of client side

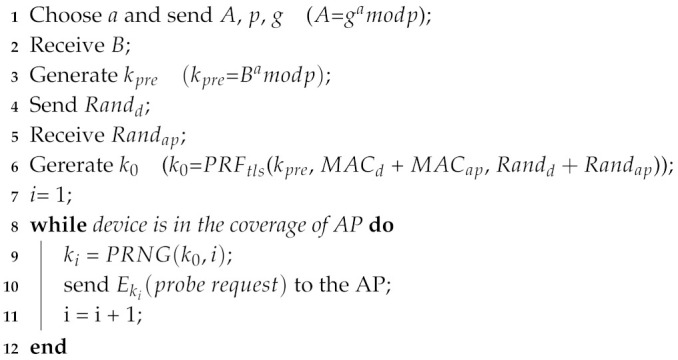



**Procedure 2:** Frame interaction procedure of AP side

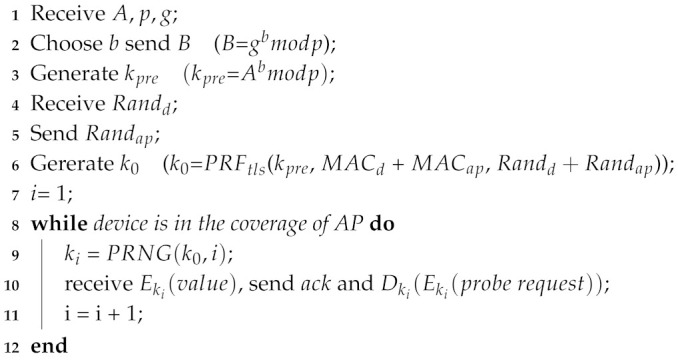



In addition, due to radio frequency interference, packet loss often happens within the Wi-Fi network. When packet loss occurs in the encrypted probe request transmission, the client is required to resend the probe request and the key generation by PRNG may be out of synchronization. Therefore corresponding strategies on the client and AP sides to synchronize key generation are proposed. In the client side, the client which fails to receive the ack reply for a long time after sending the probe reqeust, it will use the retransmitted probe request frame that was sent last time and the key will not be updated. In AP, it needs to check whether the received frame is resent by the client. When the loss of probe request frame happens, it does not affect the procedure of AP. However when the ack frame loss happens, the AP should not use the new key but AP does not know ack frame loss instantly. Therefore whether the received frame is the same as the last received one need to be checked. If they are found to be the same in the probe request, AP remains its last key as the current key.

## 6. Defense Evaluation

In this section, the experiment setup is introduced and the performance of our proposed defense mechanism is evaluated.

### 6.1. Experiment Setup

The effectiveness of the defense mechanisms should be verified by a commercial network card, but there are great difficulties in actual operation. First, sending an encrypted probe request frame in a real network enviroment requires modification of the device driver and firmware which are different among various 802.11ac devices. Furthermore, most of them are closed source softwares, which bring great difficulties to the experimental verification of the defense mechanism. Therefore, in order to verify the effectiveness of proposed defense mechanism, Wi-Fi card is configured to monitor mode which can support packet sniffing and packet injection. The packet sniffing means that the Wi-Fi card provides the function of receiving any packets sent from any transmitter and the packet injection means the Wi-Fi card is able to send customized packets out.

- **Client and AP**: Two Dell OptiPex 3600 mini Workstation is used as a client device and AP. The client device is equipped with a Wi-Fi card and the AP is equipped with two Wi-Fi cards. The three Wi-Fi cards are capable of packet sniffing and injection, which are supported by the RTL8812au driver. Specifically, two Wi-Fi cards are placed in client and AP respectively and they can realize half-duplex transmission and reception of commercial devices by switching from packet injection to sniffing. And the rest one constantly sends the beacon in AP. At the key exchange stage, the key exchange parameters of client and AP are encapsulated into assoication request and association response respectively.

### 6.2. Performance of Frame Encryption

**Figure 8:**
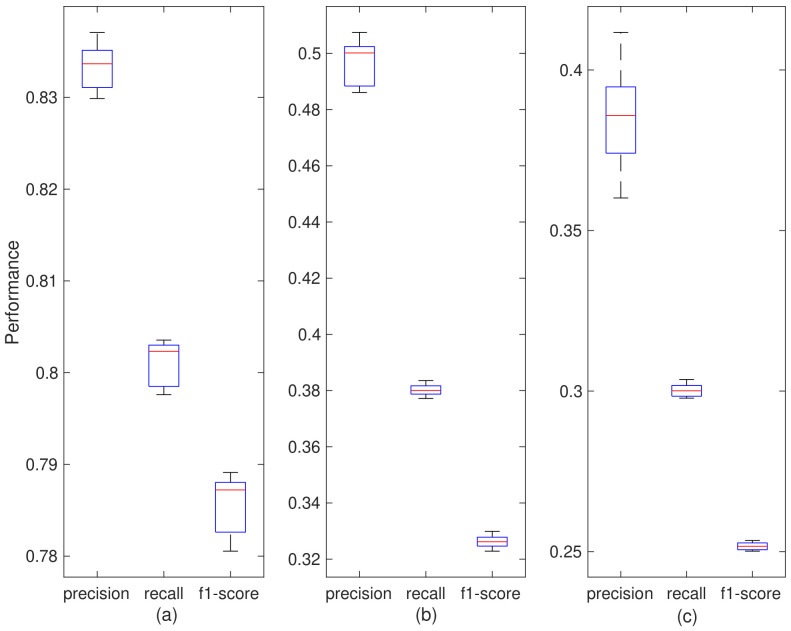
Impact of number of encrypted fields. The (**a**–**c**) individually represent the performance in three situations including encrypting the vht field only, two fields including vht and ht and all fields.

The performance of frame encryption based on the stream cipher is evaluated. In this process, PyCryptodome library is adopted to generate cryptographically secure random numbers as the secret keys to testing the performance of frame encryption. Firstly, we study how the number of encrypted fields affect the performance of device identification. Random numbers as keys are generated to encrypt the different combination of fields. The 20,000 keys as a key dataset are applied into the encryption of 20,000 probe request frames respectively. These probe request frames are selected on average from 20 device categories in Table 2, which are previously collected for device identification. As shown in Figure 8, when the vht field has been in encryption, the average precision, recall and f1-score decrease from over 99% to 83.34%, 80.12% and 78.57%. Furthermore, it can be found that all performance metrics will drop significantly as the number of encrypted fields rises, meaning that the more fields, the more features of the message are erased. As a result, there is a greatly increased chance of device misidentification of the attacker.

In addition, we also study the influence of different keys encrypting probe request on device identification. The 10 key datasets are generated and it is found that all performance metrics have low variances, especially the f1-score keeps stable. It proves that the differences of random numbers have little impact on the performance of defense mechanism and the long-term anonymity of the device type can be guaranteed.

### 6.3. Time Overhead of Defense Mechanism

**Figure 9:**
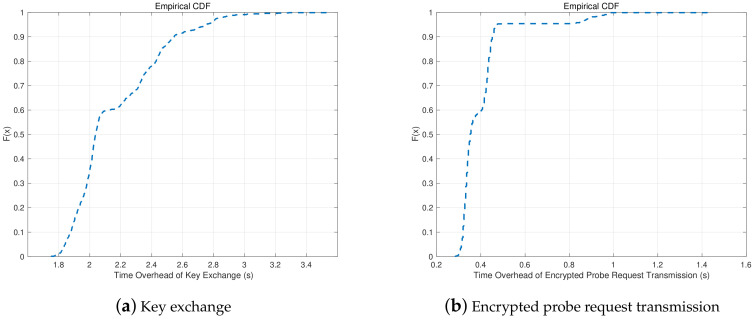
Time overhead of the two phases in the defense mechanism.

As shown in Figure 7, the defense mechanism is divided in two parts which are key exchange and encrypted probe request transimission. The time overhead of key exchange and encrypted probe request transmission are evaluated from the client side. As shown in Figure 9, the time overhead in 90th percentile is in the range between 2.4 s and 2.6 s in key exchange. In encrypted probe request transmission, the time overhead in 90th percentile is between 0.4 of one second and 0.6 of one second which is less than time overhead in key exchange. The main reason is that with the use of diffle-hellman, key exchange needs to spend more time in generating peer key than encrypted probe request transmission. However, the key exchange only occurs when client device enters into the coverage of AP, which costs a little time in the defense mechanism.

## 7. Conclusions

In this paper, a novel device identification method for attack and the corresponding defense mechanism is proposed respectively. From the perspective of attack, the 802.11ac frame is analyzed in detail. Then an attack method based on deep learning is proposed to select features automatically from 802.11ac MAC frames to identify the Wi-Fi devices. Meanwhile it has a better device identification performance than the previously proposed method. The average precision, recall and f1-score of device identification reach over 99%. The experimental result proves the implicit identifier like probe request requires protection. In response to the potential attack based on device identification, a novel defense mechanism based on the stream cipher is proposed. The original probe request is encrypted by stream cipher to preserve the privacy of the device and ensure the usability of probe request. Meanwhile the structure of probe request is preserved to prevent the attackers finding the defense mechanism in some extent. The experiment shows that the defense mechanism has a good performance. The average precision, recall and f1-score respectively decrease from 99%, 99%, 99% to 36%, 30% and 25%. In addition, the key exchange and encrypted probe request transmission in the defense mechanism take about 2 s and 0.5 of one second respectively, which do not bring too much time overhead to 802.11ac protocol.

In the future, we will use more samples and device types to prove the effectiveness of our proposed attack method. Meanwhile the influence of OS types on the probe request will be further investigated. The cause of random changes in some fields of the 802.11ac MAC frame by reserve engineering will be figured out and help us to learn reason deeply. For defense mechanism, further attempts will focus on designing a privacy-preserving metric. A privacy-preserving metric can provide our defense mechanisms with a theoretical basis. Lastly, our proposed defense mechanism will be deployed into the commercial devices in order to test the performance in a real network environment.

## Figures and Tables

**Table 1:** Physical capabilities.

Category	Physical Characteristic
Antenna	Antenna Pattern Consistency
	VHT Link Adaptation
	Multi-User (MU) Beamformer and Beamformee
	Number of Sounding Dimensions
	Beamformee STS Capability
	Single-User (SU) Beamformer and Beamformee
	Rx/Tx MCS Map
Data Rate	VHT Supported MCS Set
	LDPC
	STBC
Bandwidth	Short GI for 80 and Short GI for 160 & 80 + 80
	Supported Channel Width set

**Table 2:** List of 80211ac device types.

Index	MAC Address	Device Tuple
1	3c:37:86:1a:e8:a8	**O**:windows 7, **C**:MT7612U
2	9c:e3:3f:dc:fa:cc	**O**:ios 12.1.2, **C**:BCM4361
3	50:3e:aa:bb:d9:ce	**O**:windows 7, **D**:tp-link 1030.22.202.2018, **C**:RTL8812BU
4	f4:0f:24:1b:3c:37	**O**:macos 10.14.3, **C**:BCM43602
5	90:8d:6c:f3:cc:44	**O**:ios 12.1.2, **C**:BCM4345
6	74:da:38:ee:1e:32	**O**:windows 7, **D**:realtek 1030.21.302.2017, **C**:RTL8814AU
7	e0:dc:ff:d0:87:b7	**O**:miui 10.3.15, **C**:WCN3998
8	74:da:38:98:89:cd	**O**:windows 7, **D**:tp-link 1030.22.202.2018, **C**:RTL8812AU
9	10:5b:ad:83:aa:f1	**O**:ubuntu 16.04, **D**:ath10k, **C**:QCA9377
10	b4:6b:fc:f6:4c:fd	**O**:windows 10, **C**:Intel Dual Band Wireless-AC 8265
11	94:b8:6d:f4:39:bd	**O**:windows 10, **C**:Intel Dual Band Wireless-AC 1550
12	28:7f:cf:75:d0:e1	**O**:ubuntu 16.04, **C**:Intel Dual Band Wireless-AC 9260
13	08:be:ac:03:90:04	**O**:windows 7, **D**:Realtek 1024.2.618.2013, **C**:RTL8811AU
14	04:f0:21:48:6d:2a	**O**:ubuntu 16.04, **C**:QCA9880
15	2c:fd:a1:ce:c1:d3	**O**:windows 7, **D**:Broadcom 1.558.48.8 **C**:BCM4366
16	04:f0:21:49:07:51	**O**:ubuntu 16.04, **C**:QCA9882
17	80:19:34:6c:f2:b9	**O**:ubuntu 16.04, **C**:Intel Dual Band Wireless-AC 7260
18	a8:5e:45:46:5f:63	**O**:windows 7, **D**:Realtek 2023.28.115.2016, **C**:RTL8812AE
19	f8:28:19:6a:28:73	**O**:ubuntu 16.04, **C**:BCM4350
20	f0:03:8c:9a:2b:6b	**O**:windows 7, **D**:Broadcom 7.12.39.11, **C**:BCM4352

**Table 3:** Misidentified device type.

Device True Type	Device Predicted Type
10	13
11	10
18	8

**Table 4:** Performance of device identification.

Index	Precision	Recall	F1-Score
1	1.0000	1.0000	1.0000
2	1.0000	1.0000	1.0000
3	1.0000	1.0000	1.0000
4	1.0000	1.0000	1.0000
5	1.0000	1.0000	1.0000
6	1.0000	1.0000	1.0000
7	1.0000	1.0000	1.0000
8	0.9988	1.0000	0.9994
9	1.0000	1.0000	1.0000
10	0.9988	0.9988	0.9988
11	1.0000	0.9988	0.9994
12	1.0000	1.0000	1.0000
13	0.9988	1.0000	0.9994
14	1.0000	1.0000	1.0000
15	1.0000	1.0000	1.0000
16	1.0000	1.0000	1.0000
17	1.0000	1.0000	1.0000
18	1.0000	0.9988	0.9994
19	1.0000	1.0000	1.0000
20	1.0000	1.0000	1.0000
